# Long Terminal Repeat Retrotransposon Content in Eight Diploid Sunflower Species Inferred from Next-Generation Sequence Data

**DOI:** 10.1534/g3.116.029082

**Published:** 2016-05-25

**Authors:** Hannah M. Tetreault, Mark C. Ungerer

**Affiliations:** Division of Biology, Kansas State University, Manhattan, Kansas 66506

**Keywords:** genome size evolution, *Helianthus*, LTR retrotransposons, *Helianthus agrestis*, transposable elements

## Abstract

The most abundant transposable elements (TEs) in plant genomes are Class I long terminal repeat (LTR) retrotransposons represented by superfamilies *gypsy* and *copia*. Amplification of these superfamilies directly impacts genome structure and contributes to differential patterns of genome size evolution among plant lineages. Utilizing short-read Illumina data and sequence information from a panel of *Helianthus annuus* (sunflower) full-length *gypsy* and *copia* elements, we explore the contribution of these sequences to genome size variation among eight diploid *Helianthus* species and an outgroup taxon, *Phoebanthus tenuifolius*. We also explore transcriptional dynamics of these elements in both leaf and bud tissue via RT-PCR. We demonstrate that most LTR retrotransposon sublineages (*i.e.*, families) display patterns of similar genomic abundance across species. A small number of LTR retrotransposon sublineages exhibit lineage-specific amplification, particularly in the genomes of species with larger estimated nuclear DNA content. RT-PCR assays reveal that some LTR retrotransposon sublineages are transcriptionally active across all species and tissue types, whereas others display species-specific and tissue-specific expression. The species with the largest estimated genome size, *H. agrestis*, has experienced amplification of LTR retrotransposon sublineages, some of which have proliferated independently in other lineages in the *Helianthus* phylogeny.

Transposable elements (TEs) are DNA sequences capable of mobilizing within a host genome. Mobilization typically occurs either by physical excision-reinsertion events or through a process of replicative transposition whereby individual elements transcriptionally give rise to new copies that are reverse transcribed prior to insertion at new locations in the genome (Kumar and Bennetzen 1999; Feschotte *et al.* 2002). TEs that mobilize via replicative transposition (Class I TEs) are a major genomic component of many plant species because their mobilization involves sequence propagation and large-scale copy number increases. Because these events occur independently and at different rates and scales in the genomes of different plant lineages, even closely related species may diverge rapidly in TE content and genome size (Hawkins *et al.* 2006; Tenaillon *et al.* 2011).

The most abundant Class I TEs in plants are long terminal repeat (LTR) retrotransposons and are subdivided into superfamilies *gypsy* and *copia* (Kumar and Bennetzen 1999; Wicker *et al.* 2007). Differential proliferation and abundance of families (or sublineages) within these superfamilies are known to directly impact genome size evolution (Vitte and Panaud 2005; Hawkins *et al.* 2006; Piegu *et al.* 2006; Charles *et al.* 2008; Tenaillon *et al.* 2011; Piednoel *et al.* 2012; Kelly *et al.* 2015). Characterizing particular sublineages within superfamilies that undergo proliferation and determining patterns of proliferation events among related species can be a difficult task given sequence variation among sublineages and the difficulty of accurately estimating copy number abundance of elements within sublineages across taxa.

Advances in next-generation sequencing (NGS) approaches have greatly facilitated efforts to generate and characterize whole-genome-level sequence data (Lam *et al.* 2012) for model and nonmodel organisms alike (Kelly and Leitch 2011). Major impediments of *de novo* genome assembly of NGS data exist, however, on account of short-read lengths generated by many NGS platforms and the difficulty of assembling reads derived from genomes with a high repetitive fraction (*e.g.*, with a large TE component). NGS data nonetheless have proved extremely informative for characterization of the genomic TE content both within species and across related taxa (Tenaillon *et al.* 2011; Bonchev and Parisod 2013), and several analysis methods have been developed for such characterizations, even under scenarios of low sequence coverage (Macas *et al.* 2007; Kurtz *et al.* 2008; Novak *et al.* 2010).

Wild sunflowers in the genus *Helianthus* provide an opportune system for studies of TE proliferation dynamics and associated genome evolution (Giordani *et al.* 2014). *Helianthus* includes ∼49 species native to North America that are collectively widespread throughout the United States, southern Canada, and northern Mexico (Heiser *et al.* 1969) and phylogenetic relationships are well resolved (Rieseberg 1991; Schilling 1997; Schilling *et al.* 1998; Timme *et al.* 2007; Stephens *et al.* 2015). Genomic resources and tools are available for several *Helianthus* species (Kane *et al.* 2013) and a genome sequencing effort is underway for the cultivated sunflower *H. annuus* (Kane *et al.* 2011). Multiple ploidy levels are found within the genus (Kane *et al.* 2013) with genome size varying considerably even among species of the same ploidy (Sims and Price 1985). Genome structure and organization have been best characterized for the diploid species *H. annuus*. The genome of this species is highly repetitive, with LTR retrotransposons and their derivatives comprising >70% of nuclear DNA (Staton *et al.* 2012; Gill *et al.* 2014). Recent insertional activity of these sequences has been documented in *H. annuus* (Buti *et al.* 2011; Staton *et al.* 2012) as have patterns of tissue-specific expression (Gill *et al.* 2014). Recent and even larger-scale proliferation of LTR retrotransposons has been documented for three diploid annual *Helianthus* species derived via ancient hybridization events (Ungerer *et al.* 2006, 2009; Staton *et al.* 2009; Kawakami *et al.* 2010), with sublineages that proliferated in these species remaining active transcriptionally and expressed at higher levels when compared to the parental species from which the hybrid taxa are derived (Kawakami *et al.* 2011; Ungerer and Kawakami 2013).

In the current study we explore the contribution of LTR retrotransposons to genome size variation among eight diploid *Helianthus* species representing all four taxonomic sections based on current classification (Schilling and Heiser 1981) and an outgroup species, *Phoebanthus tenuifolius*. These eight *Helianthus* species represent much of the existing variation in diploid genome size, ranging nearly fourfold in estimated nuclear DNA content (Sims and Price 1985). We combine short-read NGS data with sequence information from a panel of *H. annuus* (common sunflower) full-length LTR retrotransposons in a *de novo* graph-based clustering approach that enables meaningful comparisons of LTR retrotransposon sublineage identity and abundances across species. We demonstrate that nuclear genome size is significantly correlated with repetitive DNA content in these species and that the species under investigation generally exhibit similar abundances of different LTR retrotransposon sublineages, suggestive of shared ancestry. We also note signatures of amplification for a small number of LTR retrotransposon sublineages in species with the largest genomes, thus identifying a contributing mechanism of genome size expansion in these species. Lastly, we highlight how graph-based clustering approaches are preferable to read-mapping-based approaches in interspecific comparative analyses of TE abundance.

## Materials and Methods

### Plant materials and DNA sequencing

Seeds of species utilized in this study were obtained from the United States Department of Agriculture (USDA) National Plant Germplasm System (http://www.ars-grin.gov/npgs/) or collected from natural populations (Table 1). Seeds were germinated in the dark on moist filter paper in Petri dishes and 2- to 3-d-old seedlings transferred to 8-inch pots with a 2:1 mixture of Metro-mix 350: all-purpose sand. All plants were grown under a 16 hr:8 hr, light:dark cycle in the Kansas State University glasshouse facility. Watering was conducted daily or as needed and fertilization with a weak nutrient solution (N:P:K = 15:30:15) was applied weekly.

**Table 1:** Study species, genome size estimates, and associated genomic data

Species	Abbreviation	Life Cycle	Accession	Paired-End Reads*^a^*	2C (pg) (SE)	Genome Coverage	Repetitive Fraction (%) (SE)
*H. praecox*	PRA	Annual	PI 435847	10,314,126	6.94 (0.10)	0.59	68.17 (0.18)
*H. annuus*	ANN	Annual	PI 468607	12,060,743	7.36 (0.12)	0.67	68.97 (0.21)
*H. cusickii*	CUS	Perennial	PI 649959	11,981,577	9.32 (0.24)	0.51	74.58 (0.18)
*H. divaricatus*	DIV	Perennial	PI 503212	6,752,840	9.41 (0.08)	0.29	69.55 (0.29)
*H. anomalus*	ANO	Annual	PI 468642	12,228,849	11.82 (0.37)	0.41	75.26 (0.19)
*H. heterophyllus*	HET	Perennial	PI 664732	11,753,278	11.82 (0.29)	0.40	71.42 (0.20)
*H. angustifolius*	ANG	Perennial	ANG-MCU*^b^*	6,837,151	12.91 (0.32)	0.21	73.38 (0.33)
*H. agrestis*	AGR	Annual	PI 468416	16,909,589	24.23 (0.84)	0.28	82.12 (0.15)
*P. tenuifolius*	PHO	Perennial	PHO-*LA**^c^*	10,971,465	13.94 (0.71)	0.31	74.08 (0.16)

a Post processing.

b Collected in Anson County, NC (M. C. Ungerer).

c Collected in Apalachicola National Forest (Loran Anderson).

Young, fully-expanded leaves from one individual per species were collected for DNA extraction and subsequent whole-genome shotgun (WGS) sequencing. All harvested tissue was flash-frozen in liquid nitrogen and stored at −80° until needed. Genomic DNA was extracted using the DNeasy Plant Mini Kit (Qiagen, Valencia, CA). Three micrograms of total DNA per species were utilized for library preparation and WGS sequencing on an Illumina HiSeq2000 platform, generating 2 × 100 bp paired-end reads. Library preparation was performed following the Tru-Seq standard protocol (Illumina Inc., San Diego, CA) with a library insert size of 350 bp. Libraries were multiplex sequenced on a single lane. Library construction and sequencing were performed at the University of Missouri DNA Core Facility, Columbia, MO (http://biotech.missouri.edu/dnacore/). Sequence data were trimmed and filtered using Trimmomatic V0.30 (Bolger *et al.* 2014) according to the following criteria: (1) adapters and barcodes removed, (2) reads <80 bases removed, (3) bases trimmed from read ends if quality <30, and (4) read ends trimmed while mean quality <25 in a 4 bp sliding window. Chloroplast reads were removed by mapping the filtered dataset to the *H. annuus* chloroplast genome (NC_007977.1) using BWA v0.7.6 (Li and Durbin 2009) with default parameters. Genomic coverage for each species was estimated using the equation Coverage = *LN*/*G* (Lander and Waterman 1988), where *L* is average read length, *N* is number of reads per species and *G* is genome length. Genome length for each species was calculated utilizing the haploid 1C value, derived from 2C data estimated by flow cytometry (Table 1), and the equation 1 pg = 978 Mb (Dolezel *et al.* 2003).

### Genome size determination

Nuclear DNA content (2C genome size) was estimated using a Guava PCA-96 microcapillary flow cytometry system (Guava Technologies, Hayward, CA). Five biological replicates were evaluated per species with a minimum of 5000 events per sample. Sample preparation for flow cytometry followed that of Kawakami *et al.* (2011). An external standard (*H. petiolaris*) was used along with the internal standard chicken erythrocyte nuclei (CEN; BioSure). Data were analyzed using CytoSoft V 2.5.4 (Guava Technologies, Haywood, CA).

### Estimation of genomic repetitive fraction based on short-read sequence data

The genomic repetitive fraction of each species was determined separately using a graph-based clustering approach developed by Novak *et al.* (2010) and implemented in RepeatExplorer (Novak *et al.* 2013) on the Galaxy Server (http://www.repeatexplorer.org/). Briefly, ∼3 M single end (R1) 100 bp reads were randomly sampled from each species (Table 1, Supplemental Material, Table S1) and clustered based on an all-by-all comparison of sequence similarity (≥90%) and overlap (≥55%). Individual clusters were identified and counted toward the genomic repetitive fraction if they contained ≥0.01% of the starting number of sampled sequences (*e.g.*, for 3 M sequences, minimum cluster size = 300 sequences). These parameter values represent default settings of RepeatExplorer. Datasets where fewer than 3M reads were sampled (Table S1) were automatically reduced by RepeatExplorer based on an initial analysis of a randomly sampled subset of reads and assessment of genome repeat structure as described in the RepeatExplorer manual. To assess potential variation in repetitive fraction estimates for a given dataset, five separate graph-based clustering analysis runs (each analysis run ≈3 M randomly sampled reads) were conducted, with means ± SE presented in the *Results*.

To assess the strength of association between genome size and repetitive fraction, Pearson product–moment correlation coefficients and phylogenetically adjusted correlation coefficients were performed in Program R (v3.2.2, R Foundation for Statistical Computing, Vienna, Austria). The phylogenetically adjusted correlations were performed using phylogenetic independent contrasts with the ‘APE’ package in R (Paradis *et al.* 2004), based on evolutionary relationships presented in Stephens *et al.* (2015). The phylogeny was truncated using the drop.tip function in APE to consist only of the species under investigation, with the exception of *H. anomalus*, which is of hybrid origin (Rieseberg 2006) and thus not included in the phylogenetically adjusted analysis.

### Clustering with full-length LTR retrotransposons from the H. annuus genome

To aid interpretation of repetitive sequence cluster identity and size across species as they pertain to LTR retrotransposons, graph-based clustering analysis runs were performed with a diverse reference panel of full-length *gypsy* and *copia* LTR retrotransposons derived from the *H. annuus* genome (Buti *et al.* 2011; Staton *et al.* 2012) (File S1). Individual elements were extracted and characterized from published BAC sequences utilizing the LTR*harvest* (Ellinghaus *et al.* 2008) LTR*digest* (Steinbiss *et al.* 2009) pipeline in *genometools* V 1.4.2. Of 110 full-length elements identified by these methods, 52 (40 *gypsy* + 12 *copia*) were identified as possessing an intact reverse transcriptase (*RT*) domain and thus retained for phylogenetic analysis based on their *RT* amino acid sequences (File S2). The majority of these full-length elements represent relatively ‘young’ copies, with insertion estimates within the last 2 million yr (Buti *et al.* 2011; Staton *et al.* 2012). Multiple sequence alignment was performed with ClustalW and phylogenetic analysis was conducted using neighbor-joining (NJ) and maximum parsimony (MP) methods in Molecular Evolutionary Genetics Analysis 4.0.2 (Tamura *et al.* 2007). The reliability of tree topologies was estimated with bootstrap replication (1000 pseudoreplicates).

Full-length elements subjected to phylogenetic analysis (*n* = 52) were subsequently converted to 100 bp kmers of sliding 85 bp overlap using a custom perl script to standardize sequence length with the Illumina-generated short-read dataset. By this method, 281 to 1073 kmers were generated per full-length element (35,488 kmers total). The ∼3 million Illumina reads per species were analyzed jointly with this collection of 100 bp kmers, which served as genomic ‘tracers’ enabling meaningful comparisons of the LTR retrotransposon content and abundance of different species’ genomes. The decision to use 85 bp overlap for adjacent 100 bp kmers for each full-length element was based on the fact that the resulting similarity (100% shared bases across overlap of 85%) exceeded considerably the RepeatExplorer parameters for sequence clustering (*i.e.*, ≥90% shared bases across overlap of ≥55%).

### RT-PCR assays

LTR retrotransposon transcriptional activity was evaluated via RT-PCR in both vegetative (leaf) and reproductive (bud) tissues from a single individual per species. For each sampled plant, leaf tissue representing the eight-leaf stage and the first bud were harvested and immediately flash-frozen in liquid nitrogen. Total RNA was extracted using TRIzol (Invitrogen, Carlsbad, CA) and purified with an RNeasy Mini Kit (Qiagen, Valencia, CA). RNA was treated with RNase-Free DNase (Qiagen, Valencia, CA) to eliminate DNA contamination. Two sublineages of *gypsy* and a single sublineage of *copia* were assayed for transcriptional activity in both tissue types for all species utilizing sublineage-specific primers targeting the *Integrase* and *RNASEH* domains of *gypsy* and *copia* elements, respectively (Kawakami *et al.* 2010; Ungerer and Kawakami 2013). RT-PCR assays were conducted using the ImProm-II Reverse Transcriptase system (Promega, Madison, WI; Table S2). RT-PCR reactions of the *actin* gene were used as positive controls for all samples. Negative control reactions were performed by withholding the reverse transcriptase enzyme. RT-PCR amplifications were conducted with an initial denaturing step of 94° for 2 min, followed by 5 cycles of 94° for 15 sec, 55° (+1.0°/cycle) for 15 sec, and 72° for 15 sec, followed by 30 cycles of 94° for 15 sec, 60° for 15 sec, and 72° for 15 sec, with a final incubation step of 72° for 5 min. Amplification products were size-separated via electrophoresis in 2% agarose gels and stained with ethidium bromide for visualization.

### Data availability

Raw sequence reads have been submitted to the NCBI Short Read Archive [SRP074507].

## Results

### Genome size and repetitive sequence content

Genome size estimates based on flow cytometry (Table 1) are largely consistent with earlier reports for overlapping *Helianthus* species (*n* = 7) obtained by Feulgen-staining (Sims and Price 1985), with the exception of *H. divaricatus*, which was estimated at 2C = 9.41 pg (±0.08) in the current study *vs.* 16.9 pg reported previously (Sims and Price 1985). Intraspecific ploidy variation in *H. divaricatus* may underlie this observation (E. Baack, personal communication), though it is generally thought to be rare in *Helianthus* (Kane *et al.* 2013). Greater variability in 2C values exists among the sampled *Helianthus* annual species (range = 6.94–24.23 pg) *vs.* perennial species (range = 9.32–12.91 pg; Table 1). With the exception of *H. agrestis*, all *Helianthus* species display 2C values lower than observed for closely related *P. tenuifolius* (2C = 13.94pg ± 0.71), a diploid species and outgroup taxon for *Helianthus* (Schilling *et al.* 1998; Timme *et al.* 2007; Stephens *et al.* 2015).

**Figure 1:**
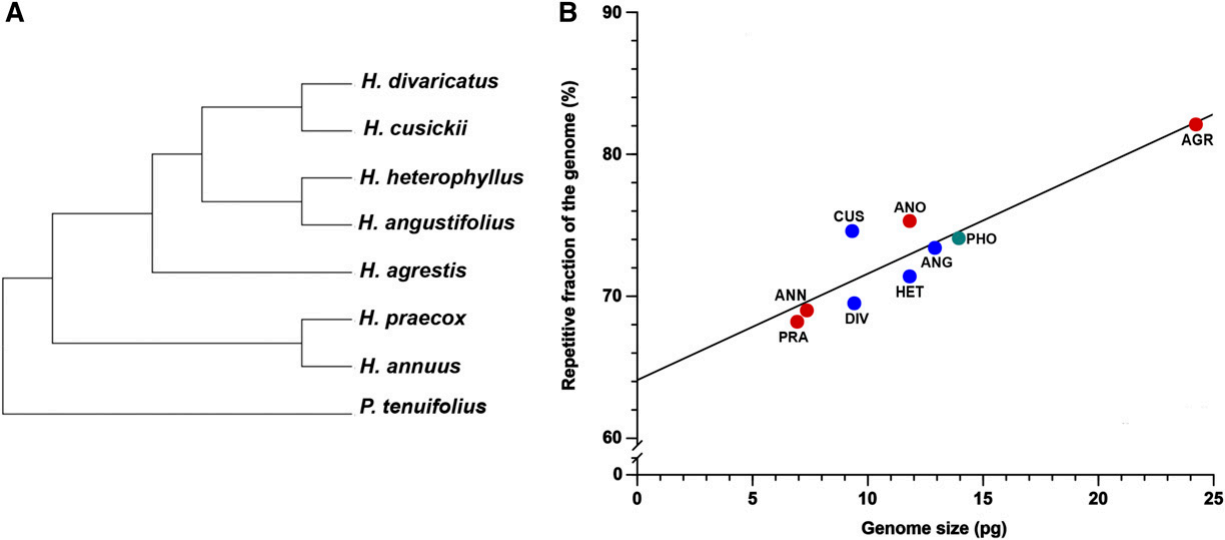
Phylogenetic relationships (A) and correlation between genome size and genomic repetitive fraction (B) for species under investigation. Phylogenetic tree is based on relationships presented in Stephens *et al.* (2015) and does not include *H. anomalus*, which is of hybrid origin (Rieseberg 2006). Genome size and genome repetitive fraction are significantly correlated: phylogenetic independent contrast analysis: *r =* 0.9041, *P =* 0.0052; unmodified analysis: *r =* 0.9121, *P =* 0.0006. Species abbreviations in (B) are as in Table 1. Red, annual; blue, perennial; teal, perennial outgroup. Values (± SE) are provided in Table 1.

The Illumina Hi-Seq platform generated ∼6.8–16.9 M paired-end reads (2 × 100 bp), post processing, for each of the eight *Helianthus* species and *P. tenuifolius*, yielding genome coverage estimates ranging from 0.21× to 0.67× (Table 1). Based on subsampling of ∼2.4–3 M single end reads per species, graph-based clustering yielded genomic repetitive fraction estimates between 68.17% and 82.12% (Table 1 and Figure 1) and these estimates are strongly correlated with estimates of genome size (phylogenetic independent contrast analysis: *r =* 0.9041, *P =* 0.0052; Figure 1).

### LTR retrotransposon contribution to genomic repetitive fraction

**Figure 2:**
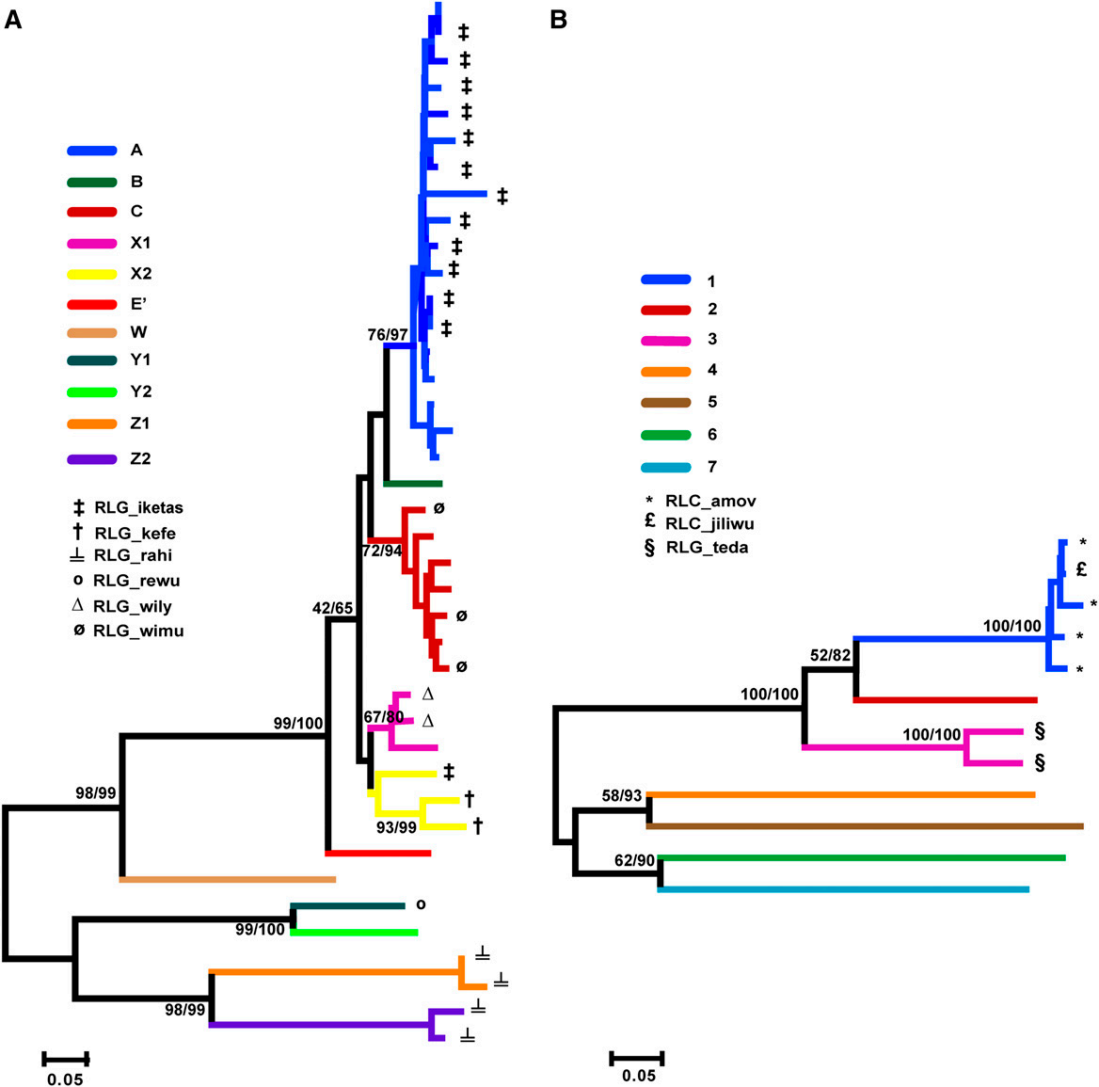
Neighbor-joining trees depicting sublineages of *gypsy* (A) and *copia* (B) elements based on 129 and 239 amino acid residues of the *reverse transcriptase* (*RT*) domain, respectively. Numbers along branches indicate bootstrap support for Maximum Parsimony/Neighbor-joining analyses. Branch colors depict different LTR retrotransposon sublineages and correspond to designations used in Ungerer *et al.* (2009) and Kawakami *et al.* (2010). Symbols at branch tips correspond to sunflower LTR retrotransposon families identified as highly abundant in *Helianthus* in Staton and Burke (2015).

To evaluate the contribution of LTR retrotransposons to the repetitive fraction of these genomes, the short-read sequence data for each species were analyzed jointly with a library of 100 bp overlapping kmers derived from 40 full-length *gypsy* and 12 full-length *copia* elements identified previously from the *H. annuus* genome (see *Materials and* *Methods*). Phylogenetic analyses based on the *reverse transcriptase* (*RT*) domains of these 40 + 12 full-length elements indicate multiple well-supported *gypsy* and *copia* sublineages (Figure 2, A and B, respectively). Comparisons of these full-length element derived *RT* amino acid sequences across sublineages for both superfamilies revealed high sequence variability, with average genetic distances ranging from 0.108 to 0.667, and from 0.318 to 0.644 in pairwise comparisons of amino acid sequences from different sublineages within *gypsy* and *copia*, respectively (Table S3). These elements are highly diverse, and represent a majority of the *gypsy* and *copia* diversity reported previously in sunflower based on sequence survey approaches (Ungerer *et al.* 2009; Kawakami *et al.* 2010) and analyses of multiple sequenced *H. annuus* BACs (Buti *et al.* 2011; Staton *et al.* 2012). Nomenclature for sublineage designations follows that reported in Ungerer *et al.* (2009) and Kawakami *et al.* (2010). Identified sublineages based on phylogenetic analyses presented herein also are largely congruent with family classification described in Staton *et al.* (2012) (Figure 2).

**Figure 3:**
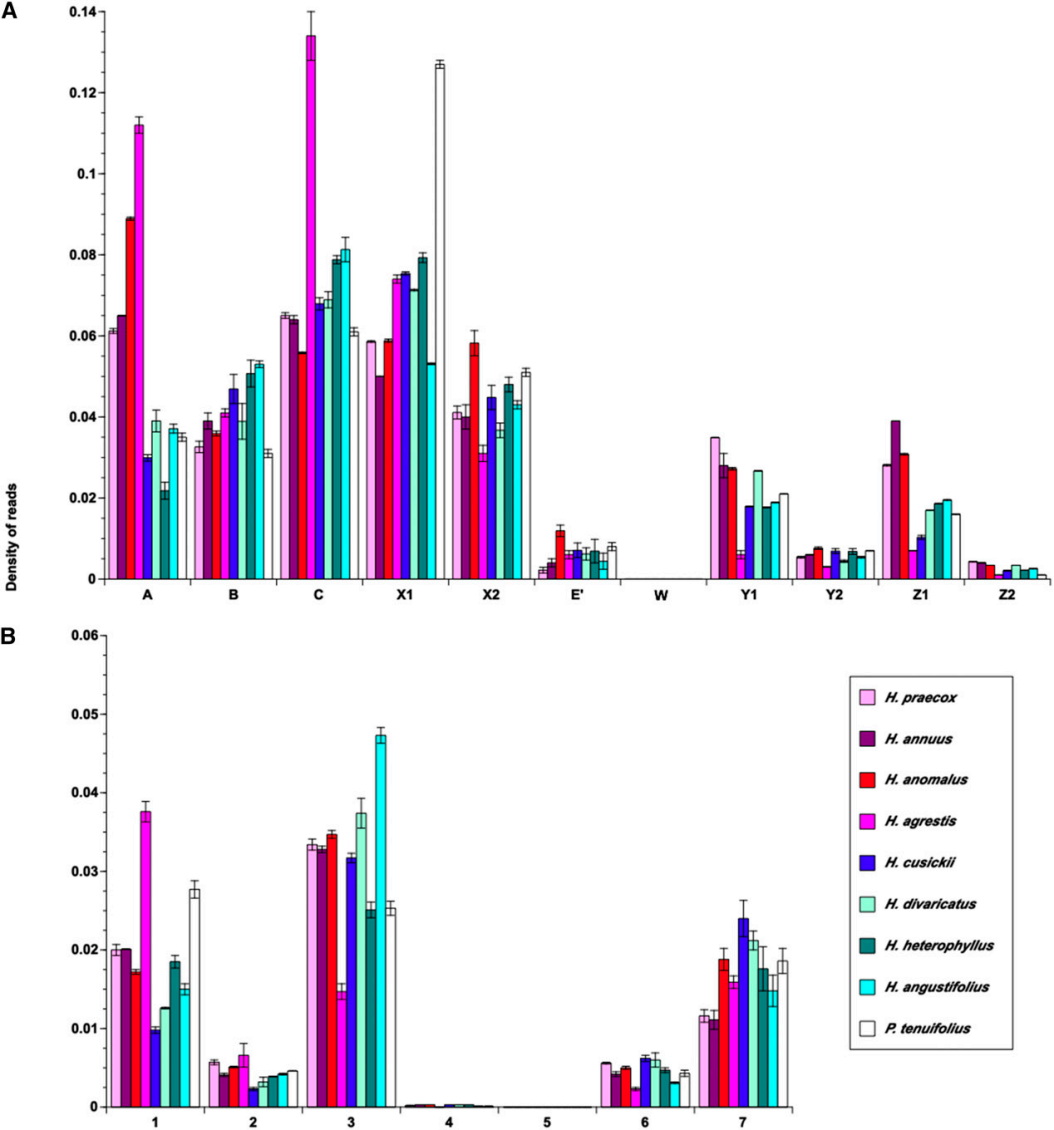
Genomic abundance of different sublineages of *gypsy* (A) and *copia* (B) elements. Shown are means (± SE) based on five graph-based clustering analysis runs for each dataset. Error bars for some histograms are too small to be seen at the resolution of this figure.

Clustering with these panels of modified full-length LTR retrotransposons allowed, for each species under investigation, assignment of short-read Illumina sequences to distinct *gypsy* and *copia* superfamilies and sublineages within these superfamilies (Figure 3, A and B). Across species, sequences derived from *gypsy* elements were 3.8- to 5.3-fold more abundant than sequences derived from *copia* elements and together sequences derived from these two superfamilies combine for between 38.3% and 49.2% of all sequences for the species assayed (Table S1). Sequences from specific *gypsy* sublineages consistently are more abundant within species’ genomes than others (*e.g.*, sublineages A, B, C, X1, and X2 *vs.* sublineages E’, Y1, Y2, Z1, and Z2; Figure 3A); these more abundant sublineages form a monophyletic group in phylogenetic analysis of *gypsy* sequences (Figure 2A).

For certain sublineages, elevated read densities were observed for some species, suggesting species-specific amplification events. For example, *H. agrestis*, the species with the largest estimated genome size and highest genomic repetitive fraction, displayed elevated read densities for two *gypsy* sublineages (A and C), indicating that proliferation of these sublineages may underlie genome expansion in this species. Similar elevated density of reads was observed for sublineage A in *H. anomalus* and sublineage X1 in *P. tenuifolius*.

Analogous patterns were observed for sublineages of *copia* elements with respect to relative abundance, with sublineages 1, 3, and 7 contributing disproportionately more, and sublineages 2, 4, 5, and 6 disproportionately less, to the genome repetitive fraction across species. Unlike observations for *gypsy* sublineages, however, the more abundant *copia* lineages are not monophyletic but rather consist of three separate, well-supported lineages in the *copia* phylogeny (Figure 2B). Elevated density was observed in *copia* sublineage 1 for *H. agrestis*, again suggestive of a role of this sublineage in genome expansion. Elevated density, though to a lesser degree, also was observed in *copia* sublineage 3 for *H. angustifolius*.

### Transcriptional activity of LTR retrotransposons in leaf and bud tissue

**Figure 4:**
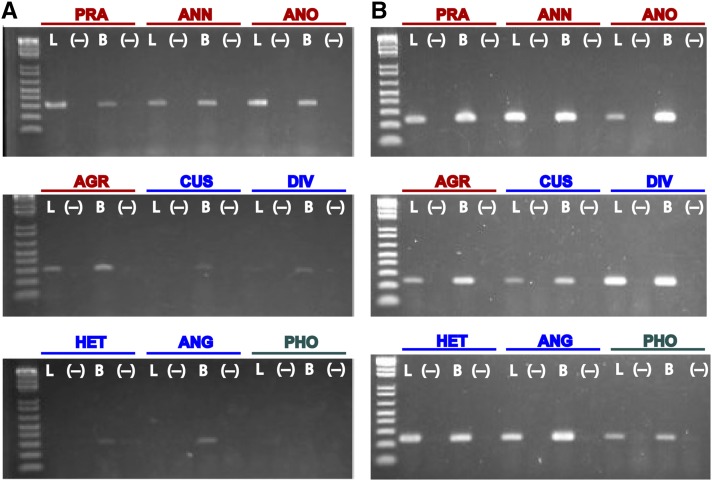
RT-PCR assays of *gypsy* sublineage A (A) and *copia* sublineage 1 (B) in leaf (L) and bud (B) tissue. Minus signs in parentheses indicate lanes with negative control reactions. Species abbreviations are as in Table 1. Red, annual; blue, perennial; teal, perennial outgroup.

Transcriptional activity of *gypsy* sublineages A and C and *copia* sublineage 1 (see Figure 2, A and B) was assayed via RT-PCR in both leaf and bud tissues for all species under investigation. Detection of transcriptional activity was variable across species and tissue types for *gypsy* sublineage A (Figure 4A), with transcripts clearly detected in both leaf and bud tissue for all annual species but less detectable in perennial species; and with more detectable expression signal in bud *vs.* leaf tissue for perennials. In contrast, transcriptional activity of *copia* sublineage 1 was equally detectable across all species and in both tissue types (Figure 4B). Transcriptional activity of *gypsy* sublineage C was not detected in any tissue type in any species (data not shown). Positive control reactions targeting *actin* expression yielded no detectable expression differences across tissue types or species (Figure S1).

## Discussion

Nuclear genome size across angiosperms varies dramatically, stretching nearly 2,400-fold between the smallest and largest documented flowering plant genomes (Leitch and Leitch 2013). Differential abundance and proliferation of TEs is now recognized as a significant contributor to genome size variation in plants, with LTR retrotransposons recognized as the most abundant and transpositionally dynamic (Hawkins *et al.* 2006; Piegu *et al.* 2006; El Baidouri and Panaud 2013). Evaluating TE diversity in organismal genomes has been revolutionized by NGS technologies that enable rapid and detailed analysis of TE composition both within and among species (Macas *et al.* 2007; Swaminathan *et al.* 2007; Wicker *et al.* 2009; Piednoel *et al.* 2012; Sveinsson *et al.* 2013; Diez *et al.* 2014; Agren *et al.* 2015; Kelly *et al.* 2015).

Here we utilized Illumina short-read sequence data coupled with sequence information from a panel of full-length *gypsy* and *copia* LTR retrotransposons obtained from the *H. annuus* genome to explore the contribution of LTR retrotransposons to genome size variation among eight diploid *Helianthus* species representing all four taxonomic sections based on current classification schemes (Schilling and Heiser 1981) and an outgroup species, *P. tenuifolius*. The species under investigation consist of both annuals and perennials, vary in genome size by ∼fourfold, yet all are diploid with a haploid chromosome complement of *n* = 17. Given that other major classes of TEs such as DNA transposons and non-LTR retrotransposons (*e.g.*, LINEs) represent a very small fraction of the sunflower genome (∼2% and 0.6%, respectively) (Staton *et al.* 2012), these other TE categories were not included in the current analyses. In addition, cluster annotation in RepeatExplorer based on the RepeatMasker Viridplantae database indicates that other repeat types (*i.e.*, low complexity repeats, simple repeats, and satellite DNA) generally are rare (<2% combined). This latter category of repeat types was thus also excluded from analysis.

### Variation in genome size

With the exception of *H. agrestis*, all *Helianthus* species investigated in the current study possess genome size estimates lower than that for the outgroup species *P. tenuifolius*. It is currently unknown whether this pattern is attributable to a general history of genome size reduction of *Helianthus* lineages, genomic expansion in *P. tenuifolius*, a combination of the two forces, or an artifact of the species sampled. Genome size reduction (DNA loss) can result from processes such as illegitimate recombination and/or unequal intrastrand homologous recombination events at the site of LTRs or interior coding regions of LTR retrotransposons (Devos *et al.* 2002; Vitte and Panaud 2003; Ma *et al.* 2004; Hawkins *et al.* 2009). Hallmarks of these events include the presence in the genome of truncated elements and solo LTRs. These hallmarks have not been thoroughly investigated in *Helianthus* species or comparatively in *P. tenuifolius* due to a lack of sufficient sequence data. Based on analyses of 21 BAC clone sequences of the common sunflower *H. annuus*, however, truncated elements and solo LTRs do not appear a common feature of the sunflower genome (Staton *et al.* 2012). Evidence for independent genome expansion in *P. tenuifolius* following divergence from *Helianthus* lineages is suggested by elevated read density for at least one *gypsy* sublineage described in the current study (Figure 3A, see also Staton and Burke 2015). Despite these observations, broader trends across the Asteraceae suggest a directional increase in abundance of the more common *gypsy* LTR retrotransposons (and accordingly in genome size) from basal to more derived lineages, the latter of which include *Helianthus* and *Phoebanthus* species (Staton and Burke 2015). As such, *Helianthus* and *Phoebanthus* species’ genomes should be considered larger and with higher copy numbers of LTR retrotransposons compared with other members of Asteraceae, at least based on the limited sampling to date.

### Clustering with panels of full-length LTR retrotransposons

A strong positive correlation was found between genome size and genome repetitive fraction, indicating an important role for repetitive DNA in underlying genome size variation in this group. Combining short-read data with sequence information from a panel of full-length LTR retrotransposons in a *de novo* graph-based clustering approach enabled meaningful comparisons of LTR retrotransposon presence and relative abundance across species. The majority of elements within this panel have estimated insertion times in the *H. annuus* genome within the last 2 million yr (Buti *et al.* 2011; Staton *et al.* 2012). As such, our analyses focus on LTR retrotransposons in *Helianthus* likely to have been active recently; more ancient elements potentially involved in older amplification events may be less well represented. Sequences derived from *gypsy* elements were observed to be 3.8- to 5.3-fold more common than sequences from *copia* elements for these species. These results are consistent with previous analyses of the *H. annuus* genome (Buti *et al.* 2011; Staton *et al.* 2012), and consistent with genomic composition analyses in other plant species where similar abundance biases have been observed (International Rice Genome Sequencing 2005; Ming *et al.* 2008; Paterson *et al.* 2009).

Our results indicate variation in abundance for different sublineages of *gypsy* and *copia* elements within genomes, but general stability in read density within a sublineage across species. Stability in read density across species is expected if most LTR retrotransposon proliferation activity occurred in the common ancestor of these species, with sequence abundances remaining relatively unchanged following subsequent cladogenesis. Elements from the most abundant sublineages of *gypsy* (*i.e.*, sublineages A, B, C, X1, and X2; Figure 3A) represent part of a larger, well-supported, monophyletic group (Figure 2A), and thus share a common evolutionary history. In contrast, *copia* sublineages with the highest read densities (*i.e.*, sublineages 1, 3, and 7) represent more distantly related and nonmonophyletic elements.

While general stability in read density within most sublineages was observed across species, exceptions to this pattern were found, most notably for three *gypsy* sublineages (sublineages A, C, and X1) and a single *copia* sublineage (sublineage 1). These exceptions were marked by higher read densities for species with larger genome size estimates, and were most apparent for *H. agrestis* and *P. tenuifolius*. These patterns likely reflect recent and lineage-specific amplifications that have contributed to genome size expansion in these species. Similar patterns have been observed in other plant genera whereby differential abundance of a small number of LTR retrotransposon lineages underlies large genome size differences among species (Hawkins *et al.* 2006; Piegu *et al.* 2006; El Baidouri and Panaud 2013). Interestingly, representative elements for two of the abundant *gypsy* sublineages (*i.e.*, RLG-iketas and RLG-wimu) and *copia* sublineage 1 (RLC-amov, RLC-jiliwu) (see Figure 2, A and B, respectively) also display signatures of recent insertional activity in the common sunflower (*H. annuus*) genome, indicating potential widespread activity throughout the genus.

Developing appropriate methods for meaningful comparisons of TE content and abundance across species genomes has become increasingly necessary as NGS technologies continue to improve and costs continue to decline. The graph-based clustering approach of short-read Illumina data combined with sequence information from a TE reference panel proved effective for interspecific analyses of sublineage identification and sequence densities in *Helianthus*, and provides a useful method when TE reference panels are available. A potential complicating factor of this method is that, due to sequence divergence among genomes, fewer sequence reads and/or sublineages might be identified in interspecific comparisons as genetic distance increases from the TE reference panel. To explore this possibility, we tested whether the density of species-specific Illumina reads clustering with *gypsy* and *copia* tracer sequences decreased with increasing genetic distance from the *H. annuus*-derived TE reference panel. We failed to find such a negative correlation (Figure S2A). Interestingly, however, a negative correlation was observed when the LTR retrotransposon panel was used as a reference in a read-mapping based approach (Figure S2B). This negative correlation persisted when mapping stringency was relaxed and greater numbers of mismatches allowed. Interspecific read-mapping to quantify TE abundances has been problematic in other species groups as well (Sveinsson *et al.* 2013), and generally should be avoided.

### Transcriptional activity of gypsy and copia

Transcriptional activity of both *gypsy* and *copia* elements has been documented previously in both cultivated (Vukich *et al.* 2009; Gill *et al.* 2014) and wild (Kawakami *et al.* 2011; Kawakami *et al.* 2014; Ungerer and Kawakami 2013; Renaut *et al.* 2014) sunflowers. In the current study we confirmed expression of these elements in two species (*H. annuus* and *H. anomalus*) and demonstrate that transcriptional activity occurs broadly across the genus. Transcriptionally active elements documented in the current study represent the same variants associated with genome expansion events documented in three sunflower homoploid hybrid species (Ungerer *et al.* 2006; Kawakami *et al.* 2010).

Transcriptional activity of *gypsy* sequences was readily detectable in both leaf and bud tissue for all annual *Helianthus* species, less detectable in bud tissue of perennial *Helianthus* species, and undetectable in leaf tissue of perennial *Helianthus* species. Although the primers used to assay for transcriptional activity were developed from *H. annuus* (an annual species), differential detection for annual *vs.* perennial species is unlikely attributable to sequence divergence with increasing phylogenetic distance from *H. annuus* given that *H. agrestis* is an independently evolved annual species and more distant genetically from *H. annuus* than the remaining *Helianthus* species under investigation (Figure S2). It is interesting to note that more detectable transcriptional activity in annual species is consistent with a higher density of reads derived from this same sublineage based on clustering analyses of genomic short-read data (Figure 3A), demonstrating a potential link between transcriptional activity and genomic abundance level of element copy number in this group of plants. Quantitative PCR experiments have confirmed such a relationship comparing annual sunflower taxa *H*. *annuus* and *H. petiolaris* with their hybrid derivative species *H. anomalus*, *H. deserticola*, and *H. paradoxus*, where higher expression was observed in species with higher copy number abundances (Ungerer and Kawakami 2013; Renaut *et al.* 2014, but see Gill *et al.* 2014). Transcriptional activity of this *gypsy* sublineage was not detected in either leaf or bud tissue of *P. tenuifolius*, indicating that expression may be restricted to within *Helianthus*.

In contrast to results for *gypsy* transcriptional activity, expression of *copia* was equally detectable among *Helianthus* annual and perennial species, across tissue types, and in *P. tenuifolius*. Read density of genomic short-read data for this same sublineage generally are comparable across annual and perennial *Helianthus* species with the exception of *H. agrestis*, for which read density is higher. More quantitative assays of transcriptional activity of both *gypsy* and *copia* elements may yield additional insights into expression dynamics of these elements across the sunflower genus. Transcriptional activity of additional sublineages of *gypsy* and *copia* have been documented previously in *H. annuus* (Gill *et al.* 2014) via RNA-seq and shown to exhibit tissue-specific expression.

### Genome expansion in H. agrestis

A notable finding of the current study is genomic amplification of LTR retrotransposon sublineages in the genome of *H. agrestis*. *H. agrestis* has a restricted geographical distribution in the southeastern United States, with populations found in central and southern Florida and in a single county in southern Georgia (Heiser *et al.* 1969). As noted above, this species is an annual, but distantly related from most other *Helianthus* annuals that form a monophyletic group and thus has independently evolved this life history form (Stephens *et al.* 2015). *H. agrestis* is atypical in being one of only two *Helianthus* species that lack a self-incompatibility system (Heiser *et al.* 1969). Genome size estimates of *H. agrestis* indicate a nuclear genome ∼1.9–3.5× larger than any other *Helianthus* species under investigation in the current study and ∼1.7× larger than that for the outgroup species *P. tenuifolius*.

Genome expansion in *H. agrestis* is associated with amplification of a small number of LTR retrotransposon sublineages, represented by two different *gypsy* sublineages and a single *copia* sublineage. Sequences from these three sublineages represent ∼28% of the *H. agrestis* genome based on our estimation procedures (Table S1). This observation is consistent with previous findings demonstrating that large interspecific variation in genome size can be attributable to a small number of LTR retrotransposon sublineages (Hawkins *et al.* 2006; Piegu *et al.* 2006; Vitte and Bennetzen 2006; El Baidouri and Panaud 2013) but contrasts with results observed for species of plants harboring some of the largest genomes (*e.g.*, *Fritillaria*) where genome composition appears to consist of highly diverse, but relatively low abundance repeat types (Kelly *et al.* 2015). As noted above, two of the three most abundant sublineages in the *H. agrestis* genome (*gypsy* sublineage A and *copia* sublineage 1) have contributed to major genome expansion events in one or more diploid hybrid *Helianthus* species (Ungerer *et al.* 2006; Kawakami *et al.* 2010), and these sublineages remain active transcriptionally across the genus. It is thus noteworthy that the same LTR retrotransposon sublineages have experienced large-scale amplification events and promoted genome expansion independently in different regions of the *Helianthus* phylogeny. The forces governing activation (and repression) of these sublineages in different *Helianthus* species’ genomes is the focus of ongoing work.

## Supplementary Material

Supplemental Material
