# Supplementary material for: Long Terminal Repeat Retrotransposon Content in Eight Diploid Sunflower Species Inferred from Next-Generation Sequence Data
Source: G3 (Bethesda). 2016 May 25;6(8):2299–308. doi: 10.1534/g3.116.029082 (PMC4978885; doi:10.1534/g3.116.029082)
Supplement: Supplemental Material [file supp_6_8_2299__index.html]

Long Terminal Repeat Retrotransposon Content in Eight Diploid Sunflower Species Inferred from Next-Generation Sequence Data — Supplemental Material 

# Long Terminal Repeat Retrotransposon Content in Eight Diploid Sunflower Species Inferred from Next-Generation Sequence Data

## Supplemental Material for Tetreault and Ungerer, 2016

**Files in this Data Supplement:**

- Figure S1 - Positive control RT-PCR assays of *Actin* in leaf (L) and bud (B) tissue. (.pdf, 130 KB)
- Figure S2 - Comparison of graph-based clustering (A) and mapping-based (B) approaches for identifying sequences derived from *gypsy* and *copia* elements across species using an LTR retrotransposon reference panel derived from *H. annuus*. (.pdf, 200 KB)
- Table S1 - Mean number of reads sampled and mean number of reads identified as belonging to different sublineages of *gypsy* (A) and *copia* (B) LTR retrotransposons based on five graph-based clustering analysis runs per dataset. (.pdf, 105 KB)
- Table S2 - Primers utilized in RT-PCR assays. (.pdf, 187 KB)
- Table S3 - Average amino acid divergence of *RT* domains within and between sublineages of *gypsy* (A) and *copia* (B) elements depicted in Figure 2. (.pdf, 175 KB)
- File S1 - Reference panel of full-length *gypsy* and *copia* LTR retrotransposons derived from *H.annuus* genome. (.zip, 150 KB)
- File S2 - Amino acid sequences for the reverse-transcriptase (*RT*) domain of 52 (40 *gypsy* + 12 *copia*) full length LTR retrotransposon elements used for phylogenetic analysis in Figure 2. (.zip, 3 KB)
