# Supplementary material for: Long Terminal Repeat Retrotransposon Content in Eight Diploid Sunflower Species Inferred from Next-Generation Sequence Data
Source: G3 (Bethesda). 2016 May 25;6(8):2299–308. doi: 10.1534/g3.116.029082 (PMC4978885; doi:10.1534/g3.116.029082)
Supplement: Supplemental Material [file supp_g3.116.029082_FigureS1.pdf]

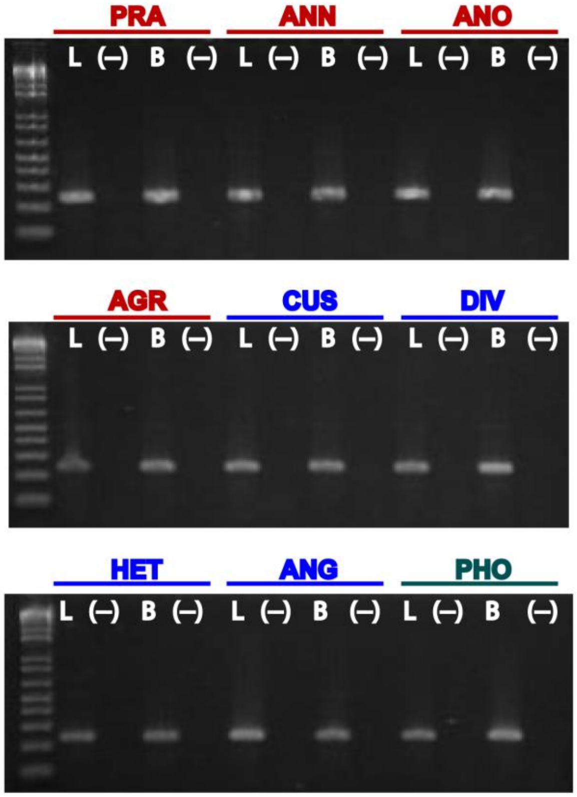

Figure S1 Positive control RT-PCR assays of *Actin* in leaf (L) and bud (B) tissue*.* Minus signs in parentheses indicate lanes with negative control reactions. Species abbreviations are as in Table 1. Red = annual, blue = perennial, teal = perennial outgroup.
