# Supplementary material for: Long Terminal Repeat Retrotransposon Content in Eight Diploid Sunflower Species Inferred from Next-Generation Sequence Data
Source: G3 (Bethesda). 2016 May 25;6(8):2299–308. doi: 10.1534/g3.116.029082 (PMC4978885; doi:10.1534/g3.116.029082)
Supplement: Supplemental Material [file supp_g3.116.029082_FigureS2.pdf]

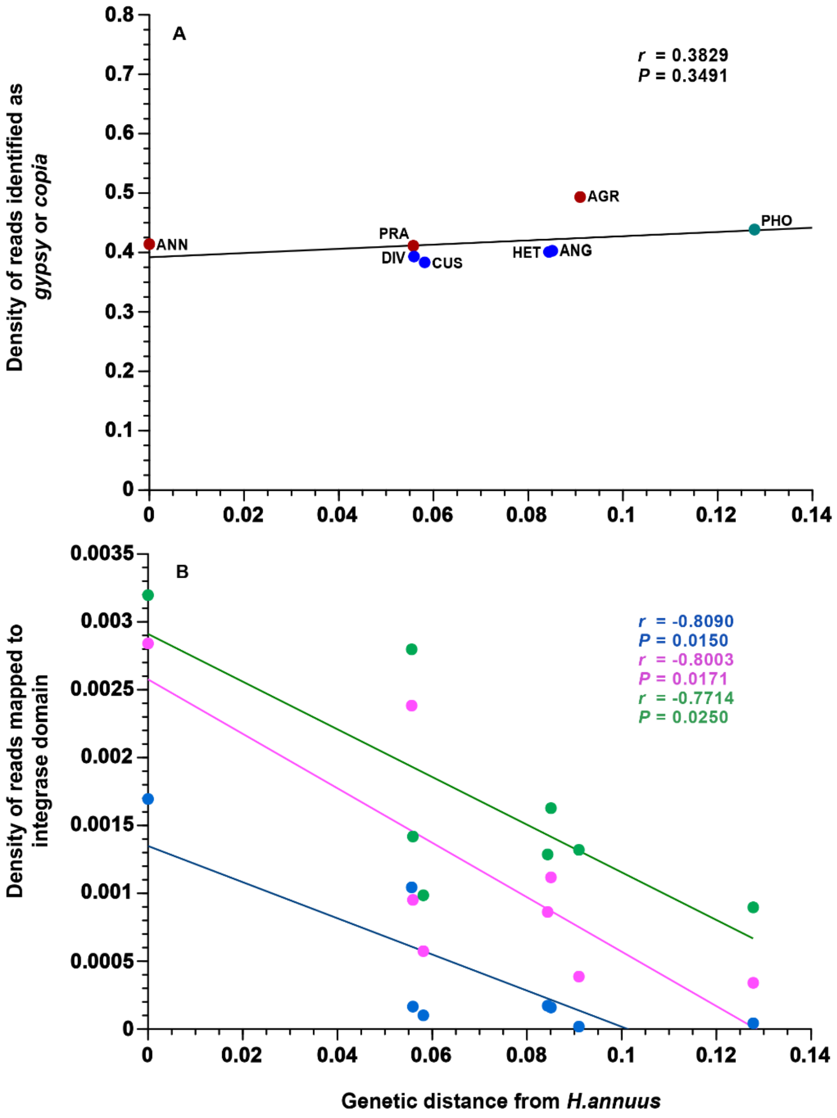

Figure S2 Comparison of graph-based clustering (A) and mapping-based (B) approaches for identifying sequences derived from *gypsy* and *copia* elements across species using an LTR retrotransposon reference panel derived from *H. annuus*. The *x*-axis depicts genetic distance from *H. annuus* for species under investigation, using 15 (randomly selected) of 170 loci used to evaluate phylogenetic relationships among diploid species of *Helianthus* (Stephens et al. 2015). *H. anomalus* was not used in these analyses because of lack of inclusion in Stephens et al. (2015). For panel (A), reads were identified as *gypsy* or *copia* sequences based on methods outlined in main text. For panel (B), 6.7 million reads from each species were mapped to the *integrase* (*INT*) domains of 52 full-length LTR retrotransposons using BWA v0.7.6. Species abbreviations are as in Table 1. For panel (A): Red = annual, blue = perennial, teal = perennial outgroup; For panel (B): blue = 4 mismatches allowed, pink = 10 mismatches allowed, green = 20 mismatches allowed.
