# Supplementary material for: Long Terminal Repeat Retrotransposon Content in Eight Diploid Sunflower Species Inferred from Next-Generation Sequence Data
Source: G3 (Bethesda). 2016 May 25;6(8):2299–308. doi: 10.1534/g3.116.029082 (PMC4978885; doi:10.1534/g3.116.029082)
Supplement: Supplemental Material [file supp_g3.116.029082_TableS1.pdf]

Table S1 Mean number of reads sampled and mean number of reads identified as belonging to different sublineages of *gypsy* (A) and *copia* (B) LTR retrotransposons based on five graph-based clustering analysis runs per dataset.

A

Species	Reads sampled	A	B	C	X1	X2	E'	W	Y1	Y2	Z1	Z2	Total reads (%)
PRA	3,000,000	183,666	97,911	195,123	175,936	123,372	6,630	19	104,742	16,169	84,280	13,038	1,000,886 (33.4)
ANN	3,000,000	196,424	115,854	193,471	149,330	119,033	13,130	13	84,352	19,193	118,365	12,245	1,021,410 (34.0)
ANO	3,000,000	266,677	107,696	167,265	176,321	174,587	35,573	12	81,569	22,902	92,417	10,187	1,135,206 (37.8)
AGR	2,368,072	265,678	96,277	317,877	175,457	72,563	14,629	1	13,164	7,951	17,342	1,445	982,383 (41.5)
CUS	3,000,000	89,677	140,613	203,565	226,226	134,301	21,219	16	53,608	20,719	30,887	6,297	927,128 (30.9)
DIV	2,542,761	99,317	98,812	175,048	181,348	93,304	15,724	6	67,856	11,148	43,100	8,686	794,349 (31.2)
HET	3,000,000	65,433	152,037	236,482	237,938	143,884	20,590	7	53,147	20,482	55,798	6,506	992,304 (33.1)
ANG	3,000,000	111,205	158,886	243,769	159,213	129,122	13,282	11	56,587	16,277	58,418	7,843	954,613 (31.8)
PHO	2,954,112	102,775	90,264	179,378	374,050	151,686	23,661	3	61,673	21,394	46,052	4,066	1,055,002 (35.7)
													8,863,281 (34.3)

B

Species	Reads sampled	1	2	3	4	5	6	7	Total reads (%)
PRA	3,000,000	60,003	16,976	100,322	734	58	16,778	34,890	229,761 (7.7)
ANN	3,000,000	60,431	12,255	98,265	768	127	12,516	33,388	217,750 (7.3)
ANO	3,000,000	51,646	15,197	104,123	847	52	14,938	56,279	243,082 (8.1)
AGR	2,368,072	89,155	15,598	34,831	62	8	5,523	37,631	182,808 (7.7)
CUS	3,000,000	29,298	6,796	95,225	773	30	18,568	72,026	222,716 (7.4)
DIV	2,542,761	32,090	8,074	95,154	666	68	15,191	53,978	205,221 (8.1)
HET	3,000,000	55,516	11,669	75,266	889	57	14,136	52,686	210,219 (7.0)
ANG	3,000,000	45,092	12,680	141,930	340	49	9,260	44,310	253,661 (8.5)
PHO	2,954,112	81,920	13,673	74,526	320	2	12,634	54,640	237,715 (8.0)
									2,002,933 (7.7)
