# Supplementary material for: Long Terminal Repeat Retrotransposon Content in Eight Diploid Sunflower Species Inferred from Next-Generation Sequence Data
Source: G3 (Bethesda). 2016 May 25;6(8):2299–308. doi: 10.1534/g3.116.029082 (PMC4978885; doi:10.1534/g3.116.029082)
Supplement: Supplemental Material [file supp_g3.116.029082_TableS2.pdf]

Table S2 Primers utilized in RT-PCR assays

Region	Forward (5'→3')	Reverse (5'→3')
gypsy sublineage A	GRTGCTTTTCCCAGCYGTTG	TCGACTCACCAAGTCTGCAC
gypsy sublineage C	AAGTCAGCKCATTTYYTACCC	TTCCARAAATGWGACGTRTATCTTAGT
copia sublineage 1	TCTCAGAACCTCGGCAATCT	GGCGAGCAAAAGAGAAAATG
actin	AGATTCCGTTGCCCTGAGGT	CTCTCTGGAGGWGCAACCAC
