# Supplementary material for: Long Terminal Repeat Retrotransposon Content in Eight Diploid Sunflower Species Inferred from Next-Generation Sequence Data
Source: G3 (Bethesda). 2016 May 25;6(8):2299–308. doi: 10.1534/g3.116.029082 (PMC4978885; doi:10.1534/g3.116.029082)
Supplement: Supplemental Material [file supp_g3.116.029082_TableS3.pdf]

Table S3 Average amino acid divergence of *RT* domains within and between sublineages of *gypsy* (A) and *copia* (B) elements depicted in Figure 2.

A

.

	A	B	C	X1	X2	E'	W	Y1	Y2	Z1	Z2
A	0.046										
B	0.118	na									
C	0.152	0.169	0.045								
X1	0.150	0.108	0.169	0.056							
X2	0.188	0.147	0.204	0.146	0.132						
E'	0.223	0.212	0.191	0.217	0.200	na					
W	0.478	0.470	0.444	0.472	0.477	0.470	na				
Y1	0.619	0.614	0.627	0.634	0.629	0.621	0.561	na			
Y2	0.627	0.621	0.628	0.636	0.621	0.614	0.568	0.235	na		
Z1	0.655	0.667	0.653	0.659	0.663	0.660	0.614	0.557	0.606	0.015	
Z2	0.647	0.622	0.638	0.619	0.648	0.629	0.633	0.561	0.572	0.445	0.045

B

	1	2	3	4	5	6	7
1	0.027						
2	0.318	n.a.					
3	0.382	0.325	0.100				
4	0.622	0.615	0.609	n.a.			
5	0.641	0.644	0.609	0.552	n.a.		
6	0.628	0.632	0.617	0.619	0.636	n.a.	
7	0.620	0.611	0.615	0.569	0.607	0.531	n.a.
